# Anti-inflammation-based treatment of atherosclerosis using Gliclazide-loaded biomimetic nanoghosts

**DOI:** 10.1038/s41598-023-41136-y

**Published:** 2023-08-24

**Authors:** Zahra Karami, Jalil Mehrzad, Mohammad Akrami, Saman Hosseinkhani

**Affiliations:** 1 Department of Microbiology and Immunology, Faculty of Veterinary Medicine, University of Tehran, Tehran, Iran; 2 Department of Pharmaceutical Biomaterials and Medical Biomaterials Research Center, Faculty of Pharmacy, Tehran University of Medical Sciences, and Institute of Biomaterials, University of Tehran and Tehran University of Medical Sciences (IBUTUMS), Tehran, Iran; 3 Department of Biochemistry, Faculty of Biological Sciences, Tarbiat Modares University, Tehran, Iran

**Keywords:** Cell biology, Chemical biology, Drug discovery, Immunology, Molecular biology, Biomarkers, Cardiology, Diseases, Molecular medicine, Pathogenesis

## Abstract

In the study, a biomimetic platform for anti-inflammatory-based treatment of atherosclerotic plaque was developed. Gliclazide (GL) as an anti-inflammasome agent was encapsulated in PLGA nanoparticles (NP), which were coated by monocyte membrane using an extrusion procedure. The size and zeta potential of the nanoghost (NG) changed to 292 and – 10 nm from 189.5 to −34.1 in the core NP. In addition, the actual size of 62.5 nm with a coating layer of 5 nm was measured using TEM. The NG was also showed a sustained release profile with the drug loading content of about 4.7%. Beside to attenuated TNFα, decrease in gene expression levels of NLRP3, MyD88, NOS, IL-1β, IL-18 and caspases 1/3/8/9 in LPS-primed monocytes exposed to NG strongly indicated remarkable inflammation control. After systemic toxicity evaluation and pharmacokinetic analysis of NP and NG, intravenous NG treatment of rabbits with experimentally induced atherosclerosis revealed remarkably less plaque lesions, foam cells, lipid-laden macrophages, and pathological issues in tunica media of aorta sections. Higher expression of CD163 than CD68 in aorta of NG-treated rabbits strongly reveals higher M2/M1 macrophage polarization. The bio/hemocompatible, biomimetic and anti-inflammatory NG can be considered as a potential platform for immunotherapy of particularly atherosclerosis in the field of personalized medicine.

## Introduction

Nowadays, nanoparticle-based treatment of inflammatory diseases has critical challenges which have attracted the attention of scientists. Among the group of inflammatory-linked diseases, atherosclerosis is a common arterial vascular injury, leading to cardiovascular complications and sometimes death. It has been reported that fat deposition in the arteries and plaque formation cause atherosclerosis, and the progression of plaque leads to fibrosis of the vessel wall. Some evidences show the plaque instability-mediated rupture results in venous thrombosis and dangerous consequences such as acute coronary syndrome (ACS) and stroke^1^.

Inflammation occurs in the early stages of atherosclerosis where immune cells migrate to the site of lipid deposition in the arteries. It is obvious that macrophages play a very important role in the development of initial plaque lesion in which monocytes differentiate into macrophages and uptake oxidized form of low density lipoproteins (ox-LDL) to form foam cells^2–5^.

Further plaque lesions in the arteries are related to secretion of pro-inflammatory cytokines and infiltration of immune cells into the plaque accumulation^5^.

It has been shown that NLRP3, a predominant component of inflammasome, is involved in the development of atherosclerosis. Indeed, the inflammasome consists of oligomers, NLRP3 sensor, caspase-1, and ASC adapter. Activation of the inflammasome complex converts pro-caspase 1 to caspase 1, converting pro-IL1 to IL1, thereby triggering acute inflammation^6–8^.

Indeed, inflammasome activation has been known as an inflammatory form of programmed cell death, called pyroptosis, where macrophages and dendritic cells play a major role instead of neutrophils^1,9–12^.

Although anti-inflammatory medications are not clinically usual to treat patients with atherosclerotic disorder, modulation of inflammatory response by anti-inflammasome agents is suggested as an alternative strategy for treatment of the atherosclerosis. Sulfonylurea derivatives, such as Gliclazide (GL), have been used to control diabetic diseases. Recently, the anti-inflammatory effects of the GL have been reported in the literature^13^, attributing the possible effects to GL antioxidant and NLRP3 inhibitory properties^14^.

Nowadays, efficient treatment of atherosclerosis has been facilitated using nanomedicine, applying nanomaterials to achieve clinical outcome^15^. Nanoparticles (NPs) are used as drug carriers for treatment and diagnosis of diseases due to their special properties including desired size, tunable shape, high solubility, good stability and penetration capability^16^.

In this area, novel biomimetic strategies have motivated scientists to fabricate bioinspired nanoscale vehicles with increased half-life as well as diminished immune response through surface functionalization. Compared to live-cell drug carriers, major advantages are mentioned for cell membrane approaches, called nanoghost (NG) and here monocytes-based nanoghosts. The cell membrane coated nanoparticles not only escape from the immune system but also cover themselves as autogenous cells. Additionally, the NG will be conducted to the target tissue using homing ligands on its surface^17^.

**Figure 1:**
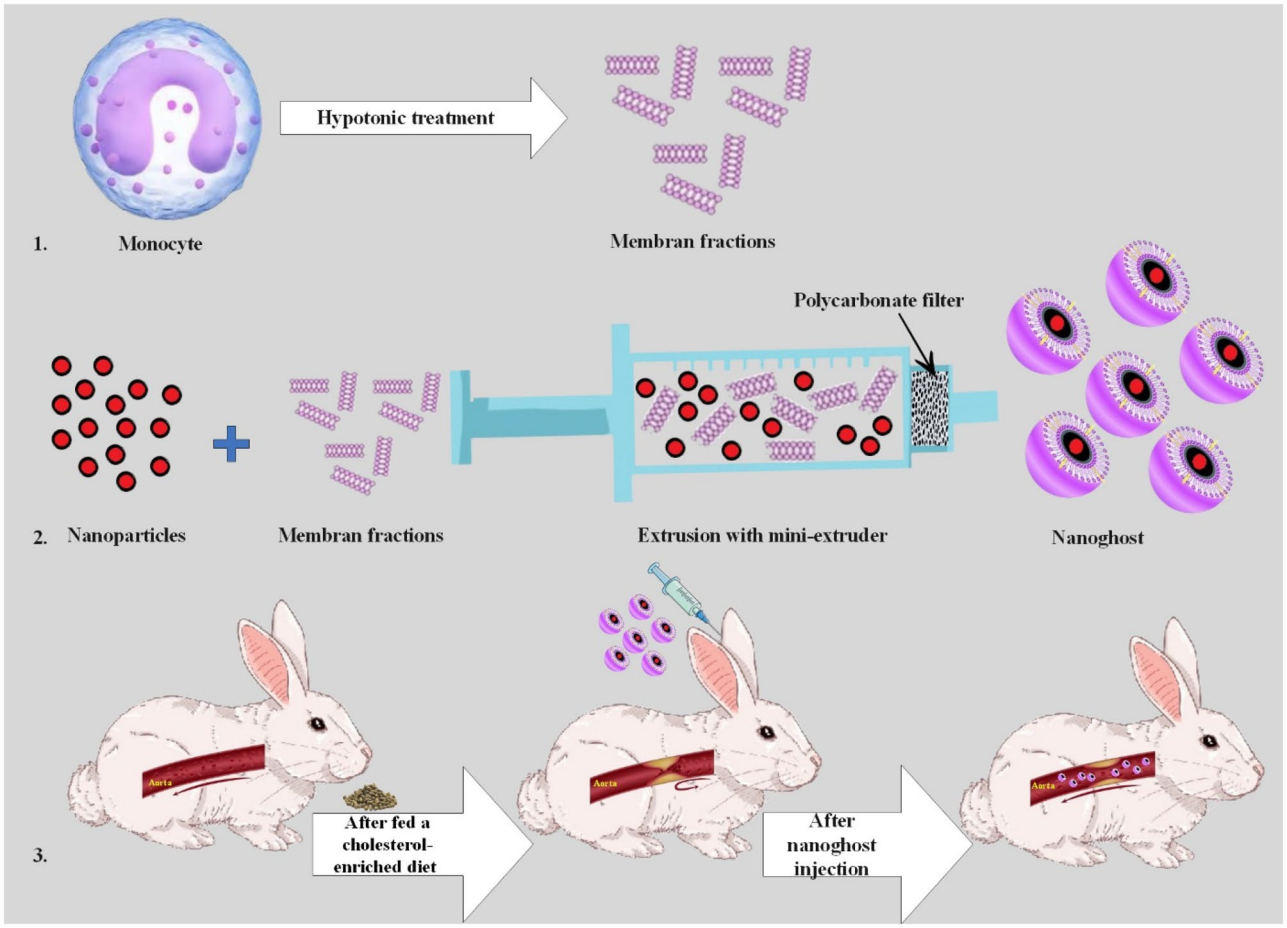
A macrophage-guided biomimetic platform designed here against atherosclerosis. A macrophage-guided biomimetic platform with anti-inflammatory agent, Gliclazide (GL), encapsulated in PLGA nanoparticles and overall coated with monocyte membrane with extrusion technique to generate pharmaceutically applicable nanoghosts for warding off atherosclerosis.

Recently, it has been reported that monocytes are a suitable platform to deliver therapeutics into the site of damaged endothelial cells in atherosclerosis, because the activated endothelial cells tether monocytes using adhesion molecules^18^. Taking the advantages of the biomimetic properties of monocyte membrane, we designed monocyte membrane coated PLGA nanoparticles using extrusion procedure after loading with GL for atherosclerosis management. The bio-mimic nanovehicle was physicochemically characterized and was assessed for hemocompatibility. The capability of the engineered NG in therapeuopreventing inflammation in the in vitro and in vivo model of particularly atherosclerosis was evaluated (Fig. 1).

## Experimental section

### Materials

GL was kindly provided by Abidi Pharma Co (Tehran, Iran). PLGA (Mw.38000-4000, 50:50) was purchased from Sigma Company (Dubai, United Arab Emirates). All reagents and solvents were purchased from Merck Company (Dubai, United Arab Emirates). The phosphatase inhibitor was supported by the Kiazist Company (Hamedan, Iran).

### Synthesis of PLGA nanoparticles

The nanoparticles were prepared by a single emulsion and solvent evaporation method according to a modified procedure^9^. Briefly, PLGA and GL were dissolved in 2 mL of dichloromethane (DCM) and then added dropwise to 20 mL aqueous phase of 1% PVA followed by probe sonication (MISONIX) at 60 amplitudes for 5 min in an ice bath. To evaporate the organic phase, the nano-emulsion was magnetically stirred for 4 h at room temperature. Finally, the emulsion was centrifuged for 15 min at 9000 rpm to precipitate nanoparticles.

### Cell culture

Accordingly^19^, isolated pure monocytes were grown in RPMI-1640 medium with 10% FBS and 1% penicillin–streptomycin. This was done in order to recheck if the quality of NGs with human pure monocytes versus U937 monocyte lines differs or is identical. The U937 monocytes (National Cell Bank of Iran, Pasteur Institute, Tehran) were then incubated at 37 °C in the presence of 5% CO_2_ until 80% confluency.

### Monocyte membrane separation

The U937 monocytes isolated in the previous step were centrifuged at 8000 rpm for 15 min followed by washing 2–3 times with PBS. Thereafter, lysis buffer (PBS, TM buffer and sucrose 0.25 M) and phosphatase inhibitor reagent were added to the cells on ice for 20 min. The cell lysate was precipitated for 35 min at 100,000 rpm with ultracentrifugation. The supernatant was discarded, and the pellets were washed with PBS. TM buffer and sucrose 0.25 M were added and stored at 4 °C.

### Synthesis of NG

The extract of U937 monocyte cell membrane was first homogenized followed by sequentially 5 times extrusion through a polycarbonate membrane of 400 and 200 nm pore sizes. The membranes were then mixed with GL loaded PLGA NP and extruded through a 200 nm polycarbonate membrane 5 times.

### Characterization of nanoparticles and NG

Hydrodynamic size and zeta potential of nanoparticle and NG were determined by zetaziser (Malvern, Nano ZS90). Furthermore, the morphology of the obtained NG was evaluated by Transmission electron microscopy (TEM).

### Assay of membrane sidedness

To verify the sidedness of the monocyte membrane for the coating of PLGA nanoparticle, the sialic acid content was assessed as an indicator of sidedness, because sialic acid distribution is asymmetric with more abundance outside of the membrane. In this regard, 100 units of sialidase (Sigma-Aldrich) was separately added to the equivalent portion of monocyte membrane as a positive control and that of PLGA NP as a negative control in parallel with the NG. After incubation for 3 h, the mixture was then centrifuged at 100,000×*g* for 1.5 h. The supernatant was finally separated for colorimetric determination of sialily groups at 549 nm.

### Drug loading estimation

About 5 mg of the GL was dissolved in 10 ml of DCM to prepare a standard stock solution. The solution was then diluted to obtain standard solutions of 15.62, 14.29, 12.82 and 12.5 mg/ml. To prepare a sample solution, freeze-dried NG was dissolved in the same solvents and vigorously shaken for an hour. The solution, which passed through the membrane filter, was measured along with standard solutions using UV–Vis spectrophotometer (JASCO-UV1500) at 243 nm (see supplementary Table 3 and supplementary Fig. 2).

### In vitro drug release

NG was dissolved in 1mL PBS buffer and transferred into a dialysis bag in a Falcon tube containing 25 mL PBS buffer medium at 37 °C, stirring at 100 rpm. 500 mL sample was taken from media at different time intervals, replaced by PBS with the same volume. The drug release percentage was determined similar to the loading estimation procedure.

### Hemolysis assay

Fresh whole blood was provided from a diagnostic lab (Pooyesh, Karaj) in a tube containing citrate. The blood sample was centrifuged at 2000 rpm to remove plasma and debris. The final RBC pellet was then diluted 3 times. 200 mL of different concentrations of NG suspension was added to the 800 mL diluted PBS suspension. PBS buffer and Triton X100 solutions were considered as positive and negative controls, respectively. The mixed solutions were then gently mixed by inversion and transferred to a water bath, shaking at 100 for 1 h at 37 °C. Subsequently, all samples were centrifuged at 10,000 rpm for 1 min, and the absorbance values of the supernatants were measured at 541 nm using UV–Vis spectrophotometer^20,21^.

### RNA extraction and RT-qPCR assays

Cells were cultured in RPMI-1640 medium as described above. After 24 h, 50 ng of LPS was added to each well and incubated for 2 h to induce inflammatory microenvironment for the cells. Thereafter, cells were treated by NG and NP (containing about 50 ng/mL of GL) and left for 24 h. Finally, the cells were collected by centrifuging at 5000 rpm for RT-PCR analysis.

Total RNA from NG-(non) treated monocytes was extracted using RNA extraction kit (Yekta Tajhiz Azma (YTA) Co., Iran Cat No. YT2551). After analyzing nanodrop and agarose gel electrophoresis, the RNA was converted to cDNA. Indeed, for cDNA synthesis, 1000 ng of total RNA was reverse transcribed into cDNA in a reaction volume of 20 μL using cDNA Synthesis Kit (YTA, Cat No: YT4500, Iran). The expression levels of some inflammatory and apoptosis-related genes for different groups were assessed by quantitative polymerase chain reaction (qPCR), using specifically two-exons junction-sequenced primers accordingly^19,22,23^. All primers were checked for the position and extra bands using Beacon Designer v8, Oligo, and NCBI, Primer-BLAST. Then, after ordering and purchasing the primers, they were diluted and used according to the manufacturer’s protocol. The primer sequences were demonstrated in supplementary Table 2.

The qPCR conditions for all genes were carried out (in triplicate) with a cycling program including holding for 10 min at 95 °C, followed by 40 cycles of 95 °C for 10 s, annealing at 61 °C for 20 s, and 72 °C for 20 s along with final extension of 72 °C for 10 min. Melting curve analysis and agarose gel electrophoresis were also performed to ascertain the specificity of the reactions or the absence of non-specific PCR products. By using GenEx 6 software^24^, data were analyzed according to the comparative Ct (2^−ΔΔCt^) method as fold change relative to the expression level of control samples^19,22,23^.

### Cytokine assay

After LPS induction and NG/NP treatment as mentioned above, 50 µL of supernatants in parallel to standards were transferred to wells in 96-well plates and incubated for 60 min under shaking at 200 rpm at room temperature. The plate was washed three times with washing solution and 50 µL of conjugated antibody was added and incubated at room temperature for 60 min on a shaker at 200 rpm. After washing, 50 µL of HRP-Avidin solution was added to all wells and incubated for 30 min at the same condition, followed by washing for 5 times. Finally, 50 µL of substrate was added to wells and incubated for 15 min before adding stop solution (25 µL). The absorbance of samples and standards was measured at 450 nm using plate reader to calculate the TNF-α levels. The sensitivity of the kit for TNF-α was < 1 pg/mL.

### In vivo toxicity

Fifteen healthy male New Zealand white rabbits (Razi Institute) with a weight average of 8200 ± 50 gr in 3 groups (PBS, NP and NG treated) were separately injected with 100 μL of NP and NG (containing 6 μg GL) as well as PBS. The treatment was followed every day for treated groups until a month. Animals were weighted every week to assess the systematic toxicity of the treatments.

### In vivo pharmacokinetic study

Animals with the same characteristics in systemic toxicity study were introduced to pharmacokinetic study. After IV injection of GL-loaded PLGA NP and NG (100 μL containing 2.88 μL of GL) into each animal, blood samples were taken at predetermined time intervals (0, 0.5, 1, 3, 6, 12, 24, 36, 48 h). The GL content was determined by HPLC. The samples were centrifuged at 12000 rpm for 8 min to separate plasma. 20 μL of plasma was added to 40 μL of Acetonitrile and mixed, centrifuged at 11,000 rpm for 4 min to separate the supernatant. The supernatant evaporated, and its residue was dissolved in the mobile phase for determination by HPLC in the same conditions as described. GL concentrations were estimated against the standard calibration curve.

The half-life (t_1/2_) and pharmacokinetic parameters of the study were statistically calculated by Excel software (version 2016).

### Animal phase

Thirty-two animals were purchased from the Razi Vaccine and Serum Research Institute (Karaj, Iran). Ethical principles of animal care and rights were observed and approved by the Research Ethics committee of the Faculty of Veterinary Medicine, University of Tehran (IR.UT.VETMED.REC.1401.006), as well as the authors. After a week adaptation period, the NG, NP and positive groups were fed with 200 g of commercial diet per day, supplemented with 2% cholesterol (w/w) for 8 weeks to induce atherosclerosis plaque. In contrast, the control group was fed with normal food. The animals were divided into four experimental groups: NG group: cholesterol-enriched fed rabbits received intravenous injections with NG; NP group: cholesterol-enriched fed rabbits treated-intravenous injections with bare drug loaded-nanoparticle; normal/control group: animals were fed with healthy/normal food without drug administration; positive control group: animals were fed a cholesterol-enriched diet without treatment.

After 8 weeks, IV administration of nanodrugs started and continued for the next 4 weeks; so, the 28th day was the endpoint of the study). Among 45 rabbits, unfortunately, 13 rabbits died due mainly to atherosclerosis complications in the induction process.

The administration was every day for NP and PBS treated groups, while it was performed every 2 days for the NG treated group (containing about 50 ng/mL) until a month.

Finally, the mix of 35 mg/kg of ketamine and 5 mg/kg of xylazine was injected intra muscularly to anesthetized rabbits. After that, animals were sacrificed to separate aorta, kidney and liver^25^. Furthermore, the biochemical analysis of cholesterol (Ch), triglyceride (TG), blood sugar (BS), Alanine amino transferase (ALT) and Aspartate amino transfer (AST) were monitored using diagnostic kits (Pars Azmoon, Iran) in Pooyesh Lab, Karaj, Iran (supporting information).

### Histopathological study

The sections were stained in Arnapat Veterinary pathology specialized (Tehran, Iran) for further macroscopic and microscopic experiments according to a procedure^26^, with a minor modification. Briefly, frozen tissue was cut into about 12 µm sections in cryostat from biopsy. Slides with section were immersed in the filtered Harris Hematoxylin for 10 s and EOSIN stain for ~ 30 s, sequentially with washing with water after each staining. Samples were then dehydrated in ascending concentrations of alcohol solutions. Finally, a coverslip was mounted onto the sections on a glass slide with a per-mount.

### Immunohistochemistry

Sections were typically stained for pan-macrophage antigens, CD68 (clone: KP1, source: Mouse Isotype: IgG1, kappa), CD14 (clone: MD85R, source: Rabbit, Isotype: IgG) and CD163 (source: Rabbit, Isotype: IgG) according to the instructions, respectively (Diagnostic Biosystems, zytomed systems, Medaysis)^27^. Furthermore, data was presented in comparison with non-immune antibody (Isotype: IgG, source: Rabbit, Non-Immune Control) based on instructions (MyBioSource company). The number of positive macrophages was counted to assess the CD68^+^ and CD163^+^ staining by a semi-quantitative estimation method^28^.

### Surface area measurement

The lesion area of plaques was quantified by OPTIKA PROView (Version X64.) which is a professional image analysis software. The total surface area of the plaque in the NG group was estimated in comparison with the positive group.

### Statistical analysis

The data were expressed in SI units and analyzed by repeated measurements ANOVA, Duncan, Spearman and T-test using SPSS software (version 12.0.4). Histochemical and immunohistochemical analyses were performed by Graphpad prism (Version 9.0.3) software. All values were expressed as mean and standard error of mean (SEM), and *P* < 0.05 was seen as statistically significant.

### Ethical statement

All protocols and procedures in the animal study and all experiments described were approved by the Research Ethics Committee of the faculty of veterinary medicine-University of Tehran (Approval ID: IR.UT.VETMED.REC.1401.006) which was in accordance to the ethical principles and the national norms and standards for conducting medical research in Iran. The protocol for hemolysis assay involving human subjects was approved by the Biomedical Research Ethics Committee (University of Tehran). To provide fresh whole blood samples, written informed consent from donors was obtained before any voluntary participation in the study. After the experiment, the blood samples were destroyed. Names and personal specifications of individual participants were not taken.

## Results

Before cell membrane isolation, the phenotype of monocytes was confirmed upon CD14 biomarker existence using flow cytometry analysis. Furthermore, the cell modes in different conditions, including cell purity and apoptosis, necrosis and cell viability were evaluated. More than about 90% of the gated isolated cell population showed a CD14^+^ phenotype (monocyte) with above 90% viability (without apoptosis and necrosis).

### Preparation and characterization of PLGA NP and NG

**Figure 2:**
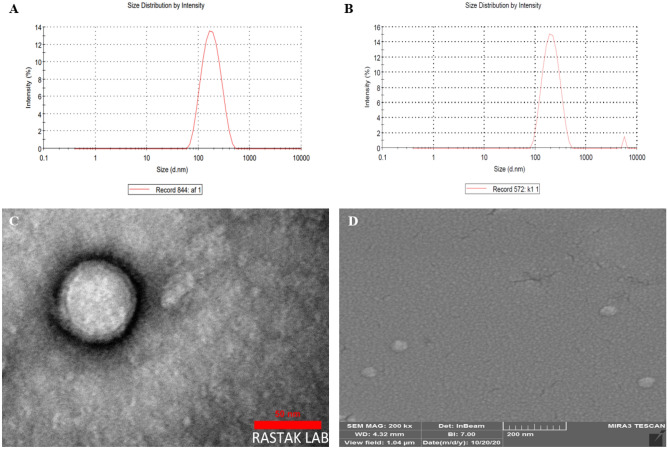
Hydrodynamic size of PLGA nanoparticles (**A**) and nanoghosts (**B**) using DLS measurement; TEM (**C**) and SEM (**D**) micrograph of drug loaded NG.

The PLGA nanoparticle from the mentioned method was successfully synthesized. As shown (Fig. 2A), the mean hydrodynamic size of 189.5 nm was measured for NP using DLS with desired size dispersity (PDI = 0.176). Furthermore, the zeta potential of the NP was − 34.1 upon measurement by a zetasizer. Coating of the PLGA nanoparticle by monocyte membrane was performed using a polycarbonate filter-based extrusion method. Compared to bare NP, the size and zeta potential of 292 nm and − 10 were obtained for cell membrane coated NP (Fig. 2B).

Furthermore, the result of the size measurement using SEM showed a diameter of about 50 nm, while diameters of 62.5 and 5 nm were estimated for NG and shell upon particle size determination by TEM, respectively (Fig. 2C,D).

According to the quantification of sialic acid content through enzymatic procedure at 570 nm, this content for the supernatant of NG was 98% in comparison with that of monocyte membrane (100%) and PLGA NP (zero) solution, indicating right-side-out membrane orientation (supplementary Fig. 2).

### Drug loading estimation and release study

**Figure 3:**
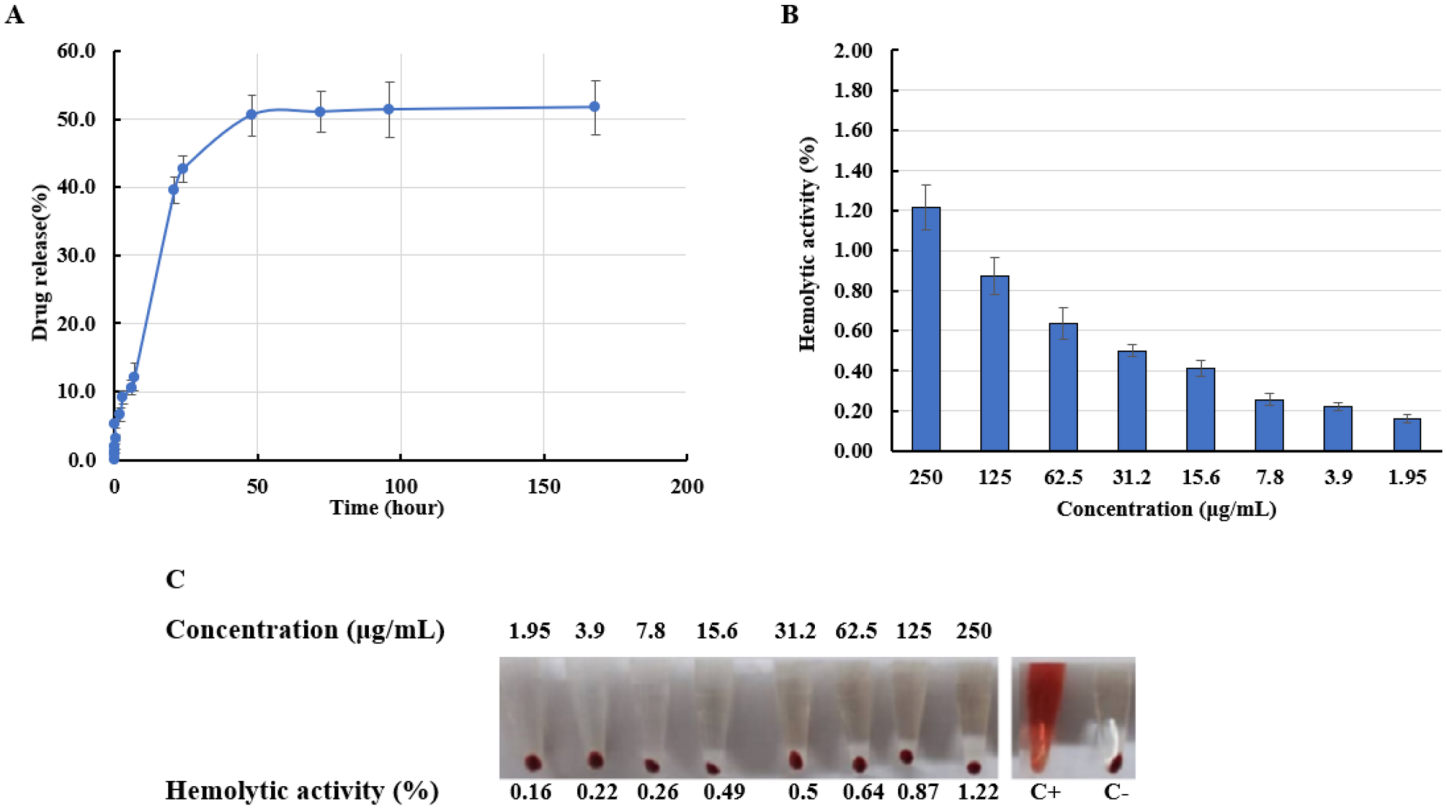
(**A**) Gliclazide release profile from nanoghosts (NG); **(B)** Hemolytic activity of drug loaded monocytes-based nanoghosts. Error bars show the standard deviation of triplicate measurements, and (**C**) demonstrates the hemolytic percentages of RBC exposed to different concentrations of NG compared to positive and negative controls.

The amount of GL in the drug loading solution as well as the dissolution samples were calculated against GL standard solutions (supplementary Table 1). For this, the calibration curve for standard solutions of GL in PBS was plotted as shown in supplementary Fig. S2. In this regard, a loading percentage of 4.68% was obtained for NG. Additionally, the burst release of GL (more than 85.3%) occurred after 24 min, which was followed by a sustained release trend until 144h (100%), as shown in Fig. 3A.

### Hemolysis assay

Hemolytic activity of the RBC exposed to different concentrations of NG showed 1.22% hemolysis in a maximum concentration of about 250 μg/ml. The lowest hemolysis was observed at a concentration lower than about 2 μg/ml (Fig. 3B,C).

### Cytokine assay

**Figure 4:**
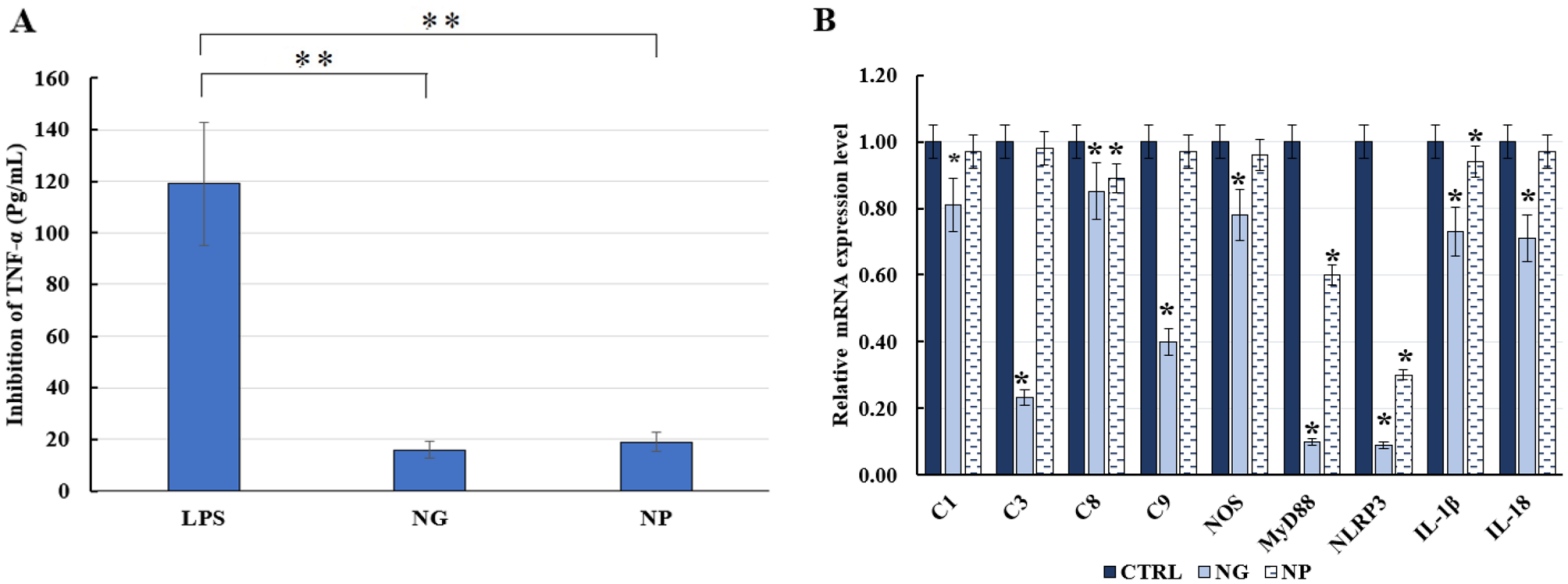
(**A**) TNFα assay of GL-loaded NG/NP on LPS-induced monocytes. Results were expressed by concentration (pg/mL) as compared with control. Data represent mean ± SD (n = 3); (**B**) Expression levels of Caspase1, Caspase3, Caspase8, Caspase9, NLRP3, NOS, MYD88, IL-1beta and IL-18 for LPS primed monocytes exposed to nanoghosts (NG) and nanoparticles (NP) compared to LPS-primed monocytes. Data represent mean ± SD (n = 3). *P-value < 0.05.

The in vitro cytokine assay in the supernatant of cell culture was examined to evaluate TNF bioactivity. The TNFα levels after NG and NP exposure were decreased to about 9.6 and 8.2 folds in comparison with LPS-induced cells as positive control (Fig. 4A).

### Key inflammatory molecules in GL-loaded NG/NP exposed monocytes at mRNA level

To investigate the effect of GL-loaded NP and NG on expression levels of genes in monocytes, compared to PBS-treated and LPS-primed cells as negative and positive controls, respectively, an RT-PCR test at mRNA level was performed.

As sequentially illustrated in Fig. 4B, the expression levels of C1, C3, C8, C9, NOS, MyD88, NLRP3, IL-1β and IL-18 was attenuated 1.23, 4.29, 1.18, 2.50, 1.05, 10.00, 11.11, 1.37 and 1.41 folds in the monocytes which were exposed to GL loaded NG, in comparison with positive control. This decrease for NP treatment was 1.03, 1.02, 1.12, 1.03, 1.04, 1.67, 3.33, 1.06, 1.03 folds for expression of the same order of genes, respectively. It should be noted that the decrease in NLRP3 level for cells exposed to NG and NP showed anti-inflammasome-mediated inhibitory of inflammation (Fig. 4B).

### Impacts of GL-loaded NG/NP experimentally induced atherosclerotic rabbit models

**Figure 5:**
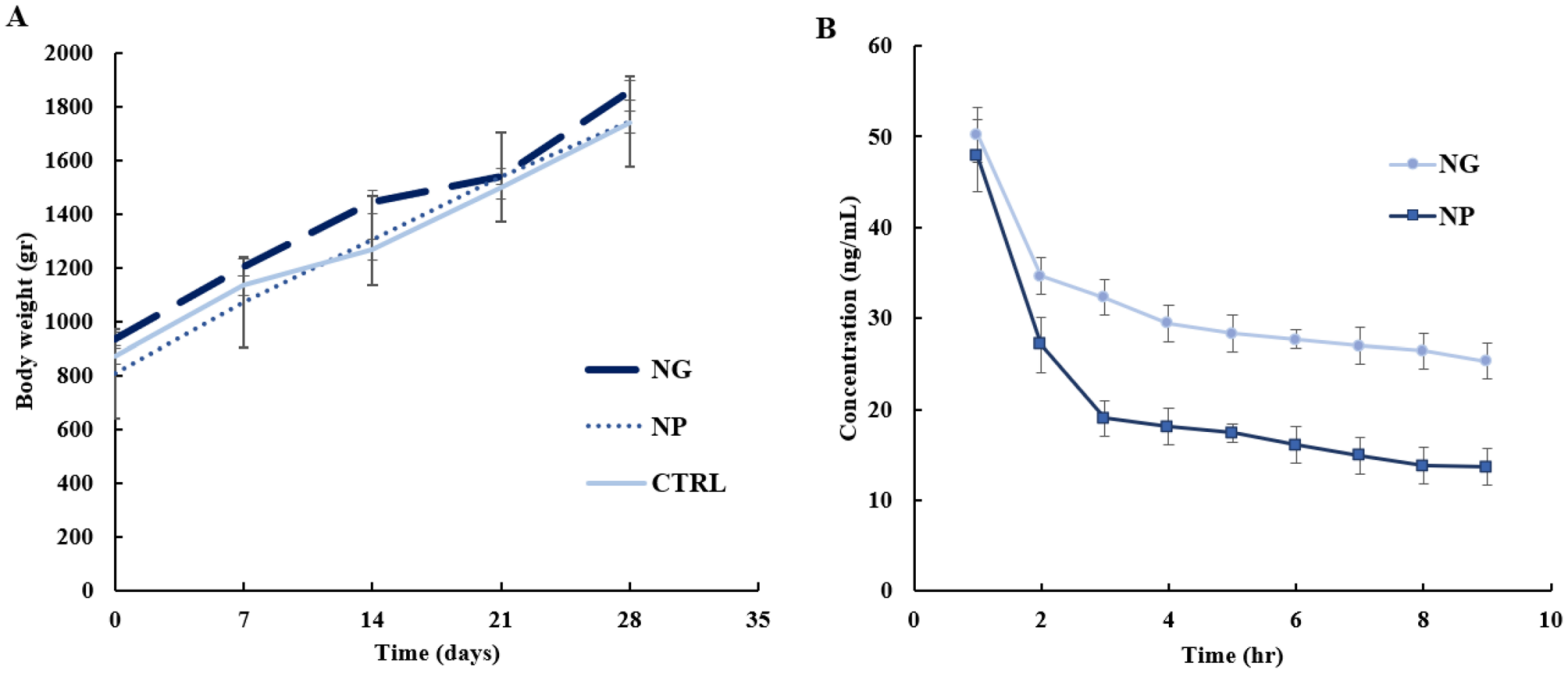
(**A**) The evaluation of systemic toxicity of nanoghost (NP) and nanoparticle (NG) (100 μL of NP and NG containing 6 μg of GL) for NG/NP treated rabbits, compared to control groups upon weigh tracking for a month (n = 8 for each group, *P*-value < 0.05). (**B**) The in vivo GL profile of drug-loaded NG and PLGA NPs in the pharmacokinetic study. 100 μL of NP and NG containing 2.88 μg of GL was injected intravenously. Data represent mean ± SD (n = 3).

Before investigating the therapeutic efficacy of NG/NP in the atherosclerotic rabbit model, the systemic toxicity experiment for administration of NG and PLGA NP was performed. The systematic toxicity upon weight monitoring for administration of NP and in NG and NP-treated groups in comparison with the control group showed no significant toxicity (Fig. 5A).

In addition, the nanoparticle dosages were determined after pharmacokinetic analysis of the administrated drug-loaded NG/PLGA NP by IV route (Fig. 5B). All parameters, including C_max_, K_e_, t_1/2_ and V_d_, were calculated. The NP administration frequency was in accordance to t_1/2_. In other words, according to the supplementary Table 3, because the calculated elimination half-life for the GL-loaded NG and NP was 48.5 h and 26.6 h, respectively, PLGA NP was administrated daily, while NG was administrated in 2 days intervals.

Biochemical analysis showed higher levels of cholesterol in the positive control rabbits exactly before medication (max = 963 mmol/L) compared to initial days (supplementary Fig. 4). Furthermore, TG levels were increased simultaneously. The glucose level was nearly constant after treatment with NG, indicating the safety of NG upon diabetic concern for animal studies at the given glucose concentration (93 mg/dl). Additionally, non-significant alteration in AST and ALT levels indicated no liver damage (supplementary Fig. 4).

**Figure 6:**
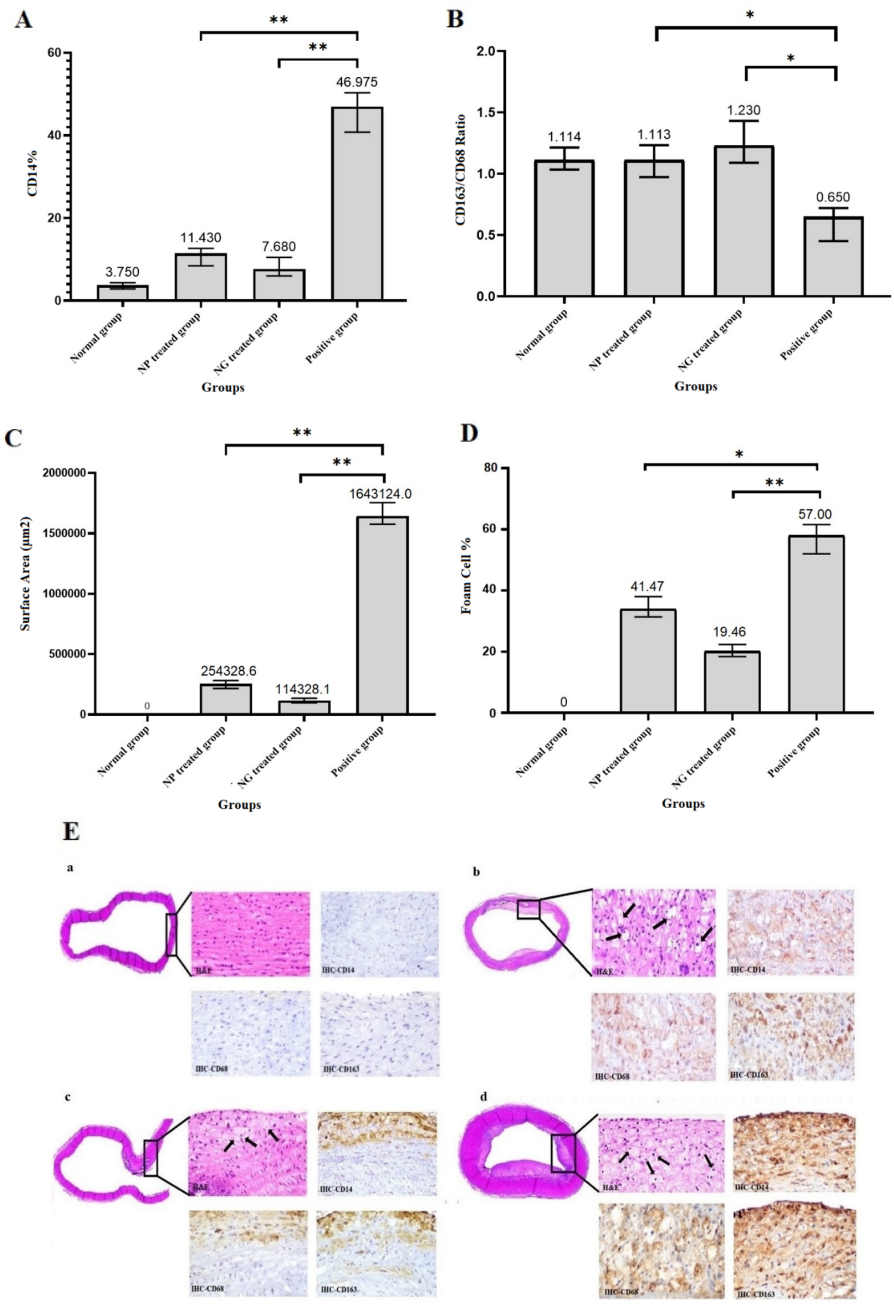
Semi-quantitative estimation of CD14 + cell percentage (**A**); CD163:CD68 ratio (**B**) through histo/immunohistochemistry analysis; Estimation of surface area (µm^2^) in intimal area of plaques using the Optika software (× 64, 4.11.18081.20201205 (**C**). Estimation of foam cell percentage (**D**) for NG/NP treated groups, compared to the normal and the positive group (*P*-values for all groups versus related positive controls were labeled with * and ** which indicate less than 0.05 and 0.01, respectively; α = 0.05). Data represent mean ± SD (n = 3). Representative hematoxylin–eosin and immuno-histochemical staining (**E**) for the aorta sections in the rabbit group fed with cholesterol-free feed/negative control (**a**); NP treated group (**b**); NG treated group (**c**) and positive group (**d**). Black arrows in Eb, Ec and Ed show the location of foam cells. 100 μL of NG and NP containing about 48 ng/mL of Gliclazide was injected into each group (n = 8) daily and every 2 days for 4 weeks, respectively.

At the endpoint of the experiment (4th week after medication), the aorta section was taken for histological analysis of atherosclerotic plaques located in the aorta root. Hematoxylin and eosin (H&E) staining as well as immunostaining using the 3, 3′-diaminobenzidine (DAB) labeled antibodies were performed to identify monocyte infiltration and macrophage phenotypes. In this regard, the expression of the biomarkers including CD14, CD68 and CD163, in the aorta sections with atherosclerotic plaque was investigated. The related statistical analysis and photographs are shown in Fig. 6. As shown in Fig. 6A, the macrophage content as CD14^+^ abundance in intima area of aorta for positive group or induced plaque group (46.98%, *P*-value < 0.01) was decreased to about 11.4% (*P*-value < 0.01) and 7.7% (*P*-value < 0.01) upon treatment with NP and NG, respectively. Furthermore, according to Fig. 6B, the CD163:CD68 ratio in NP and NG treated group was increased to about 1.1 and 1.23 respectively, compared to the positive group, which was 0.65 fold, indicating polarization of macrophages from inflammatory/M1 to anti-inflammatory/M2 phenotype. The total surface area measurement was also performed at the intimal surface to estimate the extent of atherosclerosis. The area of plaque was decreased by 14.4-fold (*P*-value < 0.01) upon treatment with NG compared to the positive group (Fig. 6C). This decrease was about 6.4-fold for the NP treated group (*P*-value < 0.01). As shown in Fig. 6D,E, the total foam cell percentage decreased to about 1.4 and 2.9-fold for the NP and NG treated group compared to the positive group (*P*-value < 0.05 and < 0.01), respectively.

H&D staining of liver and kidney of all groups are also shown in supplementary Fig. 4. No pathological issues were observed in liver and kidney sections in the rabbit group fed with cholesterol-free feed/negative control (supplementary Fig. 4A). All the mentioned pathological observations were decreased for NP (supplementary Fig. 4B) and the NG treated group (supplementary Fig. 4C). However, a degree of foam cells and interstitial lymphocytes was detected in liver sections for the NP treated group. Compared to positive control groups, no pathological issues were observed in kidney sections for NG/NP treated and negative control groups (supplementary Fig. 4B,C).

After investigation of stained liver tissues, in comparison with the normal control group, the inflammation, necrosis, number of kupffer cells, hyperplasia and interim hepatitis were observed in the liver section of positive control rabbits (supplementary Fig. 4D). This group also showed confluent necrosis with bile cholestasis.

## Discussion

In atherosclerosis, plaque instability and damage can lead to heart attack, stroke and eventually death^10^. One of the most important aspects of the disease is immunological issues and inflammation. As anti-inflammatory drugs are not given much clinical attention in patients with atherosclerosis, there is a need to develop more effective therapeutics, considering inflammatory aspects. For example, targeting NLRP3 inflammasomes in monocytes and macrophages is known as a novel therapy to inhibit inflammation^29^.

Within atherosclerotic plaque, the expression of NLRP3 inflammasome is elevated in macrophages and foam cells. Therefore, application of anti-NLRP3 inflammasome agents has been considered as an attractive approach for preventing plaque formation^30^. Nowadays, sulfonyl urea derivatives are known as effective anti-inflammasome drugs^31^. In this regard, in the study, GL was selected as an anti-inflammasome agent for atherosclerosis management^32^.

One of the major drawbacks of the drug is low solubility and uncontrolled-release. To overcome these drawbacks, GL was encapsulated into PLGA nanoparticles as an FDA approved polymer^33^. The use of the nanoparticle platform has not only solved the drug solubility issue, but also has improved inhibition of inflammation in both in vitro and in vivo study.

Due to the fact that atherosclerotic plaques have cellular and inflammatory origins and also for their treatment, the half-life of drugs in circulatory systems is important, the development of bio-mimetic based drug delivery systems has been considered by scientists. Such a drug delivery system (DDS) can not only remove obstacles resulting from immunological detection by reticuloendothelial system (RES) and subsequent fast clearance, but it can also acquire a sustained delivery aspect as well as immunological homing toward tissue. The approach eliminates the necessity to target agents and bio-conjugation. For many years, PEG has been known as a potent stealth agent for nano-drug delivery systems to ensure long circulation time of drugs by the escape from RES. Recent studies have reported the appearance of anti-PEG antibodies in the blood of a patient who experienced medication of PEG-based dosage forms^34^. Therefore, an alternative strategy is being considered due to the inefficiency of PEG coated DDS^35^. In this regard, cell membrane coated DDS as a novel bio-mimetic strategy has been developed^36,37^. Logically, the cells involved in the development of atherosclerotic plaque can be used as starting materials for bio-mimetic DDS. Monocytes and macrophages are the most important immune cells that have pathological roles in atherosclerotic plaque formation. It is known that monocytes (macrophages) migrate into the plaque formation site, converting to foam cells and triggering to undergo apoptosis upon ox- LDL uptake^18^.

Accordingly, in our study, use of cell membrane derived from the monocytes as key cells in atherosclerotic plaque formation was assumed to be the best approach for constructing the biomimetic drug-loaded PLGA nanoparticles. Such anti-inflammatory NG will be accumulated in inflamed tissues after membrane antigen associated with binding and sequestration of pro-inflammatory cytokines. Subsequently, a released anti-inflammatory drug from NG resulted in a synergistic effect against immunological aspects of atherosclerosis.

Our NG was successfully fabricated with a round morphology using an extrusion-based method applied for cell membrane homogenates and PLGA nanoparticles. An obvious shell observed using TEM in accordance with other evidence, the altered size and zeta potential of cell membrane coated nanoparticle compared to non-coated ones, confirmed cell membrane coating of PLGA nanoparticles. The smaller size of NG measured by TEM than that measured by DLS is due to the larger hydrodynamic radius in DLS measurement compared to the actual TEM size. The results of the dissolution profile showed enough GL release from NG in interval times, ensuring drug delivery as well as an anti-inflammatory effect upon homing in plaque.

Furthermore, non-significant hemolytic activity of not only the NG but also core PLGA NP even in concentrations higher than the applied one indicated excellent hemo-compatibility (< 2) of the DDS for animal study. In our study, the lowest gene expression level among genes was observed for NLRP3, indicating the powerful capability of GL-loaded nanoghost in inflammasome inhibition. Furthermore, the result together with attenuated expression of other inflammatory genes, showed that DDS has the potential to prevent inflammation in atherosclerosis plaque.

The probable weight loss of rabbits during a month of administration of NP and NG showed no decrease or stoppage in animal weight gain in comparison with the control group (PBS injection). In other words, the ascending trend in weight gain in a similar slop with the control group for PLGA NP as well as NG treated rabbits indicated no systematic toxicity upon one month continuous therapeutic injection. Accordingly, the pharmacokinetic study on the safe concentration of GL-loaded NG/NP revealed the drug half-life for dose adjustment^38^.

After animal study, the less necrotic core in aortic arch for NG-treated rabbits indicated the desired efficacy of GL-loaded NG for atherosclerosis therapy. Reduction in foam cells, lipid-laden macrophages and mineral deposits in tunica media as well as less pathological issues in liver and kidney sections proved that the drug-loaded NG has effectively inhibited plaque development in the rabbit model of atherosclerosis. Our study suggests that biomimetic NG is effective for therapy of inflammatory disease, which is in accordance with the conclusion of others^37^. Furthermore, in line with others^39^, no sign of hyperplasia in the plaque area for NG/NP treated groups confirmed the results of the study of a drug-coated balloon for atherosclerosis treatment^39^.

A higher mean count of CD14-positive cells was calculated for positive control (non-treated group fed with cholesterol enriched diet) than normal control, indicating inflammatory status of the plaque. The CD14-positive subpopulation was decreased upon NP and NG treatment, respectively, improving the inflammation aspects of atherosclerosis. The result confirmed the observation of paclitaxel therapy for cholesterol induced atherosclerosis lesions in rabbits^26^. Furthermore, the higher percentage of CD163-positive macrophages than CD68-positive ones for NP and NG treated groups, respectively, in comparison with positive control may show M1 to M2 polarization as well as anti-inflammatory phase after treatment (P values < 0.05). However, this effect was slightly stronger for the NG treated group. In comparison, the CD68/CD163 ratio for positive control indicated more pro-inflammatory phase.

The decrease in TG and cholesterol levels in blood after NG/NP exposure in comparison with the positive group showed a straight correlation with the decrease in plaque sizes as well as lipid laden foam cells. However, additional outputs for clear identification of lipid-loaded macrophages in the atherosclerotic plaque is needed to be examined.

Taken together, the novel developed DDS, GL-encapsulated NG, has potential in clinics for personalized medicine therapy of inflammatory aspects of atherosclerosis. This platform down regulates gene expression of some inflammation related molecules, decreases necrotic cores and pathological issues can increase the M2/M1 macrophages ratio. The DDS would also be a promising platform for treating other inflammatory diseases. We suppose that functionalization of the NG platform offers other advantages to accurately target inflammatory tissue, especially vascular endothelia.

## Conclusion

In summary, we successfully developed and characterized the monocyte-based NG platform in which the core GL loaded-PLGA nanoparticle was surrounded by a bio-mimetic monocyte derived cell membrane. This bio/hemocompatible nanocarrier attenuated expression of inflammatory genes like TNFα, IL-1β and IL-18. However, the lowest expression level for the NLRP3 gene introduced the GL-loaded NG as a strong anti-inflammasome platform. This NG not only decreased the inflammation, but also decreased blood levels of TG and cholesterol, total foam cells, plaque size and pathological issues in the aorta area after GL delivery. The balance of pathological issues with M2/M1 phenotype ratio after NG treatment was towards the anti-inflammatory phase. The monocyte membrane-based GL-loaded NG can be introduced as a promising biomimetic candidate for controlling inflammatory aspects and immunotherapy of atherosclerosis.

### Supplementary Information

Supplementary Information.

## Data Availability

Some datasets used and/or analyzed during the current study are available from the corresponding author on reasonable request. All data generated or analyzed during this study is included in this published article and its supplementary information files.
