# Supplementary material for: Anti-inflammation-based treatment of atherosclerosis using Gliclazide-loaded biomimetic nanoghosts
Source: Sci Rep. 2023 Aug 24;13:13880. doi: 10.1038/s41598-023-41136-y (PMC10449813; doi:10.1038/s41598-023-41136-y)
Supplement: Supplementary file 1 — Supplementary Information. [file 41598_2023_41136_MOESM1_ESM.docx]

**Supplementary information**

**Supplementary Fig. 1.** Membrane sidedness assay by measuring released sialic acid from NG after sialidase exposure. The data mean ±SD (n=3).

**Supplementary Table 1.** Drug loading calculation.

| y=0.0602X-0.4141 | |  | 0.29=0.0602X-0.4141 |
| --- | --- | --- | --- |
|  | µg/ml | µg | in 5mg=100% |
| % Loading | 11.696 | 233.92 | **4.68** |

**Supplementary Fig. 2.** The calibration curve of Gliclazide (GL) standards for estimation of drug loading and drug release. 5 mg of the GL was dissolved in 10 ml of DCM to prepare a standard stock solution. The solution was then diluted to obtain standard solutions of 15.62, 14.29, 12.82 and 12.5mg/ml. To prepare a sample solution, freeze-dried NG was dissolved in the same solvents and vigorously shaken for an hour. The solution, which passed through the membrane filter, was measured along with standard solutions using UV-Vis spectrophotometer (JASCO- UV1500) at 243 nm. The data mean ±SD (n=3).

**Supplementary Table 2:** The specific oligonucleotide sequences used in RT-qPCR assay.

| **Name** | **Seq(5'to3')** |
| --- | --- |
| Caspase1-F | TTCTGCTCTTCCACACCAGA |
| Caspase1-R | CACATCACAGGAACAGGCAT |
| Caspase3-F | ATGGTTTGAGCCTGAGCAGA |
| Caspase3-R | GGCAGCATCATCCACACATAC |
| Caspase8-F | AGAAGAGGGTCATCCTGGGAGA |
| Caspase8-R | TCAGGACTTCCTTCAAGGCTGC |
| Caspase9-F | GCAGGCTCTGGATCTCGGC |
| Caspase9-R | GCTGCTTGCCTGTTAGTTCGC |
| NLRP3-F | GGACTGAAGCACCTGTTGTGCA |
| NLRP3-R | TCCTGAGTCTCCAAGGCATTC |
| IL1B-F | AGCTTGGTGATGTCTGGTCC |
| IL1B-R | AACACGCAGGACAGGTACAG |
| IL18-F | GGAATTGTCTCCCAGTGCAT |
| IL18-R | TGGTTCAGCAGCCATCTTTA |
| MyD88-F | ACAGAGAGAGGAAGAGAG |
| MyD88-R | GAAGGAGAGAGGGAGAGA |
| NOS-F | GCTCTACACCTCCAATGTGACC |
| NOS-R | CTGCCGAGATTTGAGCCTCATG |
| GAPDH-F | CGCTCCTGGAAGATGGTGATGG |
| GAPDH-R | GTATTGGGCGCCTGGTCACC |

**Supplementary Table 3.** The pharmacokinetic parameters of Gliclazide-loaded Nanoghost/PLGA nanoparticles. The HPLC data was calculated as a mean of a triplicate experiment.

| **Parameter** | **NG** | **NP** |
| --- | --- | --- |
| **K_e_** | 0.0 | 0.0 |
| **t_1/2_** | 48.5 | 26.6 |
| **AUC** | 3356.6 | 1837.5 |
| **V_d_** | 59.8 | 59.8 |


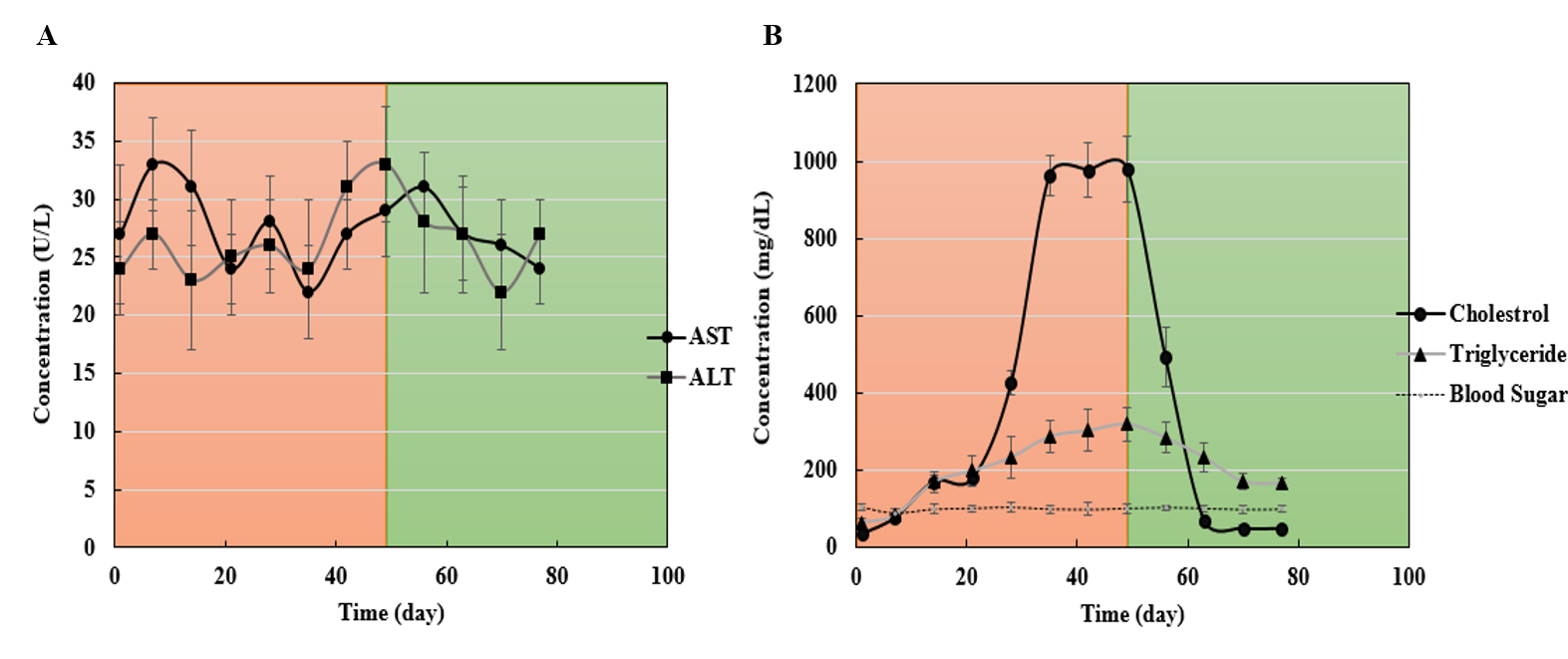


**Supplementary Fig. 3.** Biochemical analysis of experimentally plaque-induced groups in rabbit model of atherosclerosis before (pink area) and after treatment (green area) with Gliclazide loaded nanoghost (n=8 for negative control group and n=8 for each positive/NG/NP group). During the experiment, the serum levels of liver enzymes of AST (Aspartate Amino Transferase) and ALT (Alanine Amino Transferase) (**A**) as well as Cholesterol, Glucose and Triglyceride (**B**) were measured every 10 days. The data are reported as mean ±SD (n=3).


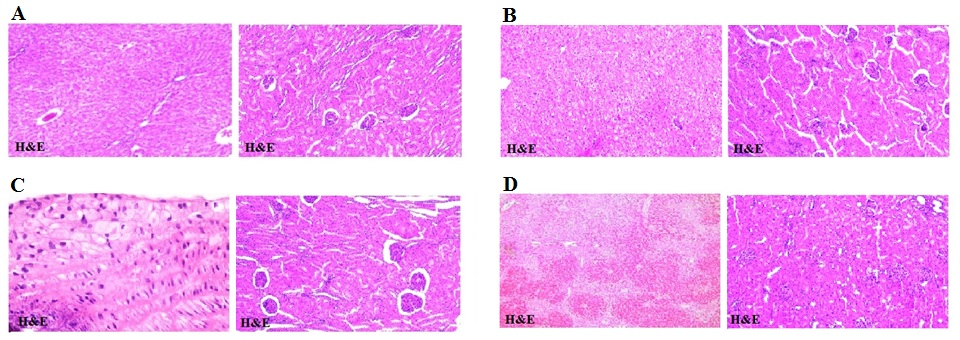


**Supplementary Fig. 4.** Representative H&E staining of liver (left side) and kidney (right side) sections, in rabbit group fed with cholesterol-free feed/negative control (A); NP treated group (B); NG treated group (C) and positive group (D). 100μL of NG and NP containing about 48ng/mL of Gliclazide was injected into each group (n=8) daily and every 2 days for 4 weeks, respectively.
